# Solute Transporter OCTN1/Slc22a4 Affects Disease Severity and Response to Infliximab in Experimental Colitis: Role of Gut Microbiota and Immune Modulation

**DOI:** 10.1093/ibd/izae135

**Published:** 2024-06-29

**Authors:** Federica Del Chierico, Letizia Masi, Valentina Petito, Valerio Baldelli, Pierluigi Puca, Roberta Benvenuto, Marco Fidaleo, Ivana Palucci, Loris Riccardo Lopetuso, Maria Emiliana Caristo, Cinzia Carrozza, Maria Cristina Giustiniani, Noritaka Nakamichi, Yukio Kato, Lorenza Putignani, Antonio Gasbarrini, Giovambattista Pani, Franco Scaldaferri

**Affiliations:** Immunology, Rheumatology and Infectious Diseases Research Area, Unit of Human Microbiome, Bambino Gesù Children’s Hospital, IRCCS, Rome, Italy; Department of Medical and Surgical Science, Digestive Disease Center (CeMAD) Translational Research Laboratories, Fondazione Policlinico Universitario “A. Gemelli” IRCCS, Rome, Italy; Department of Medical and Surgical Science, Digestive Disease Center (CeMAD) Translational Research Laboratories, Fondazione Policlinico Universitario “A. Gemelli” IRCCS, Rome, Italy; Immunology, Rheumatology and Infectious Diseases Research Area, Unit of Human Microbiome, Bambino Gesù Children’s Hospital, IRCCS, Rome, Italy; Department of Medical and Surgical Sciences, UOS Inflammatory Bowel Diseases, Center for Diseases of Digestive System (CeMAD), Fondazione Policlinico Universitario A. Gemelli IRCCS, Rome, Italy; Department of Translational Medicine and Surgery, Catholic University of the Sacred Heart, Rome, Italy; Department of Pathology, Fondazione Policlinico Universitario A.Gemelli IRCCS, Rome, Italy; Department of Biology and Biotechnologies Charles Darwin, Università La Sapienza, Rome, Italy; Department of Basic Biotechnological Sciences, Intensive and Perioperative Clinics, Fondazione Policlinico Universitario A.Gemelli IRCCS, Rome, Italy; Institute of Microbiology, Catholic University of the Sacred Heart, Rome, Italy; Department of Medical and Surgical Sciences, UOS Inflammatory Bowel Diseases, Center for Diseases of Digestive System (CeMAD), Fondazione Policlinico Universitario A. Gemelli IRCCS, Rome, Italy; Department of Medicine and Ageing Sciences, “G. d’Annunzio” University of Chieti-Pescara, Chieti, Italy; Center for Advanced Studies and Technology (CAST), “G. d’Annunzio” University of Chieti-Pescara, Chieti, Italy; Cen.Ri.S Policlinico Gemelli UNICATT Rome, Rome, Italy; Department of Clinical Biochemistry, Laboratory and Infectious Science, Fondazione Policlinico Universitario A.Gemelli IRCCS, Rome, Italy; Department of Pathology, Fondazione Policlinico Universitario A.Gemelli IRCCS, Rome, Italy; Faculty of Pharmacy, Takasaki University of Health and Welfare, 60 Nakaorui-machi, 370-0033, Takasaki, Gunma, Japan; Faculty of Pharmacy, Kanazawa University, Kakuma-machi, Kanazawa 920-1192, Japan; Unit of Microbiology and Diagnostic Immunology, Unit of Microbiomics and Immunology, Rheumatology and Infectious Diseases Research Area, Unit of Human Microbiome, Bambino Gesù Children’s Hospital, IRCCS, Rome, Italy; Department of Medical and Surgical Science, Digestive Disease Center (CeMAD) Translational Research Laboratories, Fondazione Policlinico Universitario “A. Gemelli” IRCCS, Rome, Italy; Department of Translational Medicine and Surgery, Catholic University of the Sacred Heart, Rome, Italy; Department of Medical and Surgical Science, Digestive Disease Center (CeMAD), Fondazione Policlinico Universitario “A. Gemelli” IRCCS, Rome, Italy; Department of Translational Medicine and Surgery, Catholic University of the Sacred Heart, Rome, Italy; Department of Medical and Surgical Sciences, UOS Inflammatory Bowel Diseases, Center for Diseases of Digestive System (CeMAD), Fondazione Policlinico Universitario A. Gemelli IRCCS, Rome, Italy; Department of Translational Medicine and Surgery, Catholic University of the Sacred Heart, Rome, Italy

**Keywords:** OCTN1, gut microbiota, dextran sodium sulphate colitis

## Abstract

**Background:**

Inflammatory bowel diseases are chronic disabling conditions with a complex and multifactorial etiology, still incompletely understood. OCTN1, an organic cation transporter, could have a role in modulating the inflammatory response, and some genetic polymorphisms of this molecule have been associated with increased risk of inflammatory bowel diseases. Until now, limited information exists on its potential in predicting/modulating patient’s response to therapies. The aim of this study was to evaluate the role of OCTN1 in modifying gut microbiota and mucosal immunity in response to infliximab therapy in murine colitis.

**Methods:**

A dextran sodium sulphate model of colitis was used to assess the clinical efficacy of infliximab administered intravenously in *ocnt1* gene knockout mice and their C57BL/6 controls. Stool, colon, and mesenteric lymph node samples were collected to evaluate differences in gut microbiota composition, histology, and T cell populations, respectively.

**Results:**

*Octn1* ^*-/-*^ influences the microbiota profile and is associated with a worse dysbiosis in mice with colitis. Infliximab treatment attenuates colitis-associated dysbiosis, with an increase of bacterial richness and evenness in both strains. In comparison with wild type, *octn1*^*-/-*^ mice have milder disease and a higher baseline percentage of Treg, Tmemory, Th2 and Th17 cells.

**Conclusions:**

Our data support the murine model to study OCTN1 genetic contribution to inflammatory bowel diseases. This could be the first step towards the recognition of this membrane transporter as a biomarker in inflammatory conditions and a predictor of response to therapies.

**Key Messages**

- What is already known? OCTN1 is an organic cation transporter with an established role in trafficking drugs, metabolites, and antioxidants; moreover, the association between its L503F variant and IBD has been largely confirmed in CD and UC. Despite this, scanty information is available on how this transporter contributes to IBD pathogenesis, and the mechanistic underpinnings remain elusive.
- What is new here? Our study aims to explore the role of OCTN1 in modulating gut microbiota, mucosal immunity, and response to infliximab in a murine model of colitis.
- How can this study help patient care? *octn* genotyping in IBD patients combined with microbiota and immune profiling could help monitor disease progression and predict individual response to therapy.

## Introduction

Inflammatory bowel diseases (IBDs) are multifactorial, chronic, gastrointestinal inflammatory disorders, in which genetic predisposition, environmental factors, and an altered microbiota composition play an essential role in initiating and perpetuating the disease. In particular, an atypical immune response to the autologous gut microbiota triggers IBD in genetically susceptible individuals.^1^ Indeed, existing theories propose that pathologic imbalances in the gut microbial communities, referred to as dysbiosis, activate the immune mucosal response, leading to chronic intestinal inflammation. The hallmark of active IBD is an aberrant mucosal infiltration by innate immune cells (primarily neutrophils, macrophages, and dendritic cells) and adaptive immune cells (T and B cells). Effector CD4+ T cells (Th1, Th2, Th17) are critical in the defense against pathogens, whereas regulatory T cells (Treg) play a significant role in limiting the expansion and overactivity of CD4+ effector T cells.^2–4^ Inflammatory bowel disease seems to be due to either an excessive activation of effector T cells and/or an alteration of T cell-mediated tolerance mechanisms, the latter through defects in the development of Treg or impairment of their immunosuppressive properties.^2^

The association between the L503F nonsynonymous variant of the organic cation transporter OCTN1/*SLC22A4* and IBD is well established in both Crohn’s disease (CD)^5^ and ulcerative colitis (UC).^5,6^ OCTN1 is expressed ubiquitously; and in the intestine, it is located at the brush border membrane of enterocytes where it contributes to the intestinal absorption of several dietary substrates.^7^ The OCTN1 ability to interact with hexogen molecules other than human metabolites suggests its possible role in mediating host/microbiota cross-talk. In fact, among substrates of OCTN1, the antioxidant ergothioneine^8^ is produced by fungi and bacteria and has a protective role in intestinal inflammatory status.^9^ However, there are no definitive evidences that indicate that gut microbiota can produce ergothioneine,^10^ but gut bacteria could compete with the host for using ergothioneine. Moreover, OCTN1 expression is induced by interleukin (IL)-1β and tumor necrosis factor (TNF)-α after a liposaccharide- (LPS-) stimulation.^11^ Consequently, the increase of OCTN1 enables the entrance of ergothioneine in the intracellular space, causing the suppression of IL-1β expression via its antioxidant action.^8^ However, despite this proposed positive role of OCTN1 and ergothioneine, something is still controversial. For instance, OCTN1 gene expression and ergothioneine concentration are reportedly higher in inflamed ileal mucosa of CD patients and intestinal tissues of DSS-treated mice compared with healthy controls.^12^

Apart from ergothioneine, the reported transport capacity of OCTN1 towards acetyl-choline^13^ and sperimine^14^ add further layers of complexity to the enigmatic linkage between this solute carrier and IBD. In fact, acetyl-choline has well established non-neuronal immunomodulatory roles via auto and paracrine loops, especially in the intestine^15^; on the other hand, polyamine metabolism affects T helper function, and its disruption leads to intestinal inflammation in mice.^16^ Thus, OCTN1 function may impinge on the microbe-host interaction and, by extension, shape microbiota composition and susceptibility to gut inflammation by regulating mucosal immunity.

The *octn1* 503F variant, compared with the wild-type 503L, has increased affinity for ergothioneine, acetylcholine, carnitine, and other endogenous or microbial substrates.^5,12^ Although a mechanistic connection between this variant and IBD is far from being established, loss of OCTN1 transporter specificity may decrease the uptake of physiologic compounds, while increasing the absorption of potential toxins, such as putrescine, derived from bacterial catabolism.^5^ Together with the reduction of fatty acids beta-oxidation due to low levels of carnitine in the intestinal epithelium, these changes may explain the role of *octn1* variants in colitis.^17^

The TNF-α inhibitors, like infliximab (IFX), are therapeutic options for IBD patients, with proven efficacy in moderate to severe UC with an inadequate response to conventional glucocorticoid treatment.^18^ Nevertheless, some patients do not respond or lose sensitivity to IFX during maintenance therapy,^19^ in a fashion that is difficult to predict based on the current knowledge.

In the present study, we tested the hypothesis that OCTN1 genetic asset could modify gut microbiota composition, immune reactions, and disease response to IFX therapy in dextran sodium sulphate (DSS)-induced colitis in mice. This model recapitulates histopathological characteristics of human IBD^20^ and has been successfully employed to investigate the pathogenesis of the disease. We focused on differences in microbiota composition and T cell populations between *octn1*^-/-^ and wild type (WT) mice, in the presence or absence of induced colitis. Moreover, we characterized microbial and immune profiles during the anti-TNFα treatment to address the role of OCTN1 in the individual response to this therapy.

## Materials and Methods

### Mice

We used C57BL/6 female mice of 8 weeks of age for the experimental procedures. The *ocnt1* knockout mice (*octn1*^*−/−*^) were generated as described in Kato et al^9^ and backcrossed for at least 6 generations in the C57BL/6 background. Animals were maintained with free access to food and water. All the experiments were approved by the local ethical committee and the Italian Ministry of Health.

### Experimental Acute Colitis

Eight-week-old wild type and *octn1*^*-/-*^ C57BL/6 mice were fed for 7 days with 2.5% Dextran sodium sulphate (DSS) in drinking water (MP Biomedicals, Aurora, OH, USA) provided ad libitum, then allowed to recover for a further 7 days (Supplementary Figure 1). Mice were divided into 2 groups: at day 3 of DSS treatment, the first group received IFX intravenously (5 mg/Kg); the second group received a saline solution. A second intravenous IFX treatment was administered at day 5 of DSS treatment at the first group, while in the second one a saline solution was administered. After 7 days, the DSS treatment was interrupted, and for a further 7 days mice were monitored until they were humanely killed after 14 days starting DSS treatment.

On alternate days, body weight, fecal consistency, and the presence of occult fecal blood were monitored in order to calculate the Disease Activity Index (DAI).^21^

Fecal samples were collected at T0 (pre-DSS), T1 (after 3 days of DSS administration, no IFX), T2 (after 7 days of DSS and IFX treatment), T3 (end of recovery week, 14 days after starting DSS), and immediately frozen at −80°C until analysis. The colon was collected and then fixed in 4% formalin and embedded in paraffin. Rachmilewitz and Geboes score were used for histology assessment.

### Bacterial DNA Purification, Amplification, and Sequencing

DNA was extracted from 200 mg of fecal sample, by QIAmp Fast DNA stool mini kit (Qiagen, Germany). Bacterial libraries were obtained by the amplification of the V3-V4 region of the 16S rRNA gene using primers reported in the MiSeq rRNA Amplicon Sequencing protocol (Illumina, San Diego, CA).^22^ Negative and positive controls were used to monitor and exclude eventual external and internal contaminations. The sequencing was performed on an Illumina MiSeq platform (Illumina, San Diego, CA, United States), where paired-end reads of 300 base-length were generated.

Raw sequences were joined, trimmed, filtered for chimera sequences, and matched against the Greengenes 13.8 database^23^ by QIIME software.^24^ MicrobiomeAnalyst^25^ was used to calculate α- and β-diversity and statistical tests (Mann-Whitney *U*, Kruskal-Wallis, Benjamini-Hochberg, DESeq2 tests) on taxa relative abundances. Data were scaled with total sum scaling method.

PICRUSt v1.1.0 tool was applied to predict metagenome functional content from 16S rRNA gene surveys.^26^ The resulting function prediction was analyzed by HUMAnN2 v0.99 program to obtain the KEGG (Kyoto Encyclopedia of Genes and Genomes) pathways (http://huttenhower.sph.harvard.edu/humann2).^27^

### Isolation and Culture of Mesenteric Lymph Node Cells

Mesenteric lymph node (MLN) cells were removed aseptically at the time of sacrifice, and cells were gently dispersed through a 100- and 70-μm cell strainer to obtain single-cell suspensions. Note that 1 × 10^6^ resulting cells were cultured in Roswell Park Memorial Institute 1640 Medium with 10% calf serum, 1% P/S, and 1% glutamine for 48 hours in the presence of 1 μg/mL of anti-CD3/CD28 monoclonal Ab.^28^ Mesenteric lymph node cells were placed in a culture medium supplemented with 1x GolgiStop for 4 hours at 37°C. After the incubation period, the cells were collected for flow-cytometry assay, as described later on.

### Flow Cytometry

To identify viable cells, cultured MLN cells were stained with Fixable Viability Stain 780 (BD Horizon) to determine cell viability, followed by incubation for 10 to 15 minutes at room temperature protected from light. Cells were then stained with antimouse surface CD25, CD4, CD45, CD62L, CD44, and CD3 Abs for 30 minutes at 4°C in the dark. After that, cells were washed with permeabilization buffer and stained with antimouse intracellular FOXP3, IL17A, IFNϒ, IL6, TNFα, and IL4 Abs for 50 minutes at 4°C in the dark. Since in mice with spontaneous ileitis similar to Crohn’s disease,^29^ a proportion of Treg lost CD25, in addition to the classic marking for the Treg, we have analyzed the Treg populations not expressing CD25 and expressing interleukin-17 (FOXP3+, CD4+, CD25- and IL17+) and the Treg expressing interleukin-17 (FOXP3+, CD4+, CD25+ and IL17+). Flow-cytometric acquisition was performed on a BD FACSLyric Flow Cytometer instrument for lymphocytes T evaluation. Data were subsequently analyzed using FlowJo V10 software.

### Multiplex Immunofluorescence Staining

The Opal 4-color immunohistochemistry (IHC) kit (PerkinElmer, Waltham, USA, Cat. No. NEL820001KT) was employed for the 4-color multiplex immunolabeling on 5-micron sections from FFPE (Formalin-Fixed, Paraffin-Embedded) tissue samples of mouse. The chosen panel includes antibodies against LY6G, CD4, CD8, and CD11b.

Detailed information regarding the antibodies include LY6G (ab236132;Abcam, EPR22909-135 clone, 1:1000 dilution, opal 520, ER2 30’ retrieval and incubation time); CD4 (AB288724;Abcam, RM1013 clone, 1:1000 dilution, opal 570, ER1 30’ retrieval and incubation time); CD8 (AB217344;Abcam, EPR21769 clone, 1:2000 dilution, opal 620, ER1 30’ retrieval and incubation time); CD11b (ab133357;Abcam, EPR1344 clone, 1:3000 dilution, opal 690, ER1 30’ retrieval and incubation time).

Slides corresponding to 1 tissue section per analyzed antibody were baked 3 hours at 65°C in a laboratory oven. Then, the staining process was conducted using the automated immunostainer, Leica BOND RX (Leica Microsystems, Milton Keynes, UK).

Fluorophores and 4′,6-diamidino-2-phenylindole (DAPI) were prepared according to the guidelines provided by the manufacturer. The image was acquired with Vectra Polari Automated Quantitative Pathology Imaging System (Akoya Biosciences), and the analysis was performed with Akoya Bioscience’s inForm software. All data processing and visualization procedures were executed on R (version 4.2.2), facilitated by the phenoptrReports and phenoptr packages.

### IFX Quantification in Blood Samples

Serum levels of IFX in control and DSS-treated mice were measured by ELISA (Sanquin, the Netherlands) on 10 microliters of defibrinated plasma, according to the manufacturer’s recommendations.

### Immunohistochemistry for OCTN1

Briefly, sections were automatically stained by immunostainer BOND MAX III (Leica). Sections (~4 μm) of formalin-fixed paraffin-embedded tissues were deparaffinized and rehydrated through a series of alcohol/water solutions, followed by blocking of endogenous peroxidases with a 3% hydrogen peroxide solution. Tissues were subjected to heat-induced antigen retrieval using a citrate buffer ph6 for 10 minutes. An antimurine OCTN1 rabbit antiserum^9^ (Dil.1:300) was applied to target tissues for 60 minutes. Detection of immunocomplexes was accomplished using a Universal HRP-Polymer detection system (Bond polymer refine detection). In a final detection step, 3,3ʹ-diaminobenzidine (DAB) was applied for visualization. Slides were briefly counterstained in a modified Mayer’s hematoxylin.

### Statistical Analysis

The Student’s *t* test (2-tailed) was applied for comparisons of colitis and immune features. Provided the data fulfilled the assumptions for parametric statistics, comparison between more than 2 groups was carried out by 2-way ANOVA. All data were expressed as mean ± SD. *P* values < .05 were considered statistically significant. All statistical analyses were performed used GraphPad Prism (version 9, GraphPad Software, San Diego, CA, USA).

## Results

### *Octn1* ^-/-^ Mice Display a Better Course of Colitis Compared With Wild Type Controls

**Figure 1.**
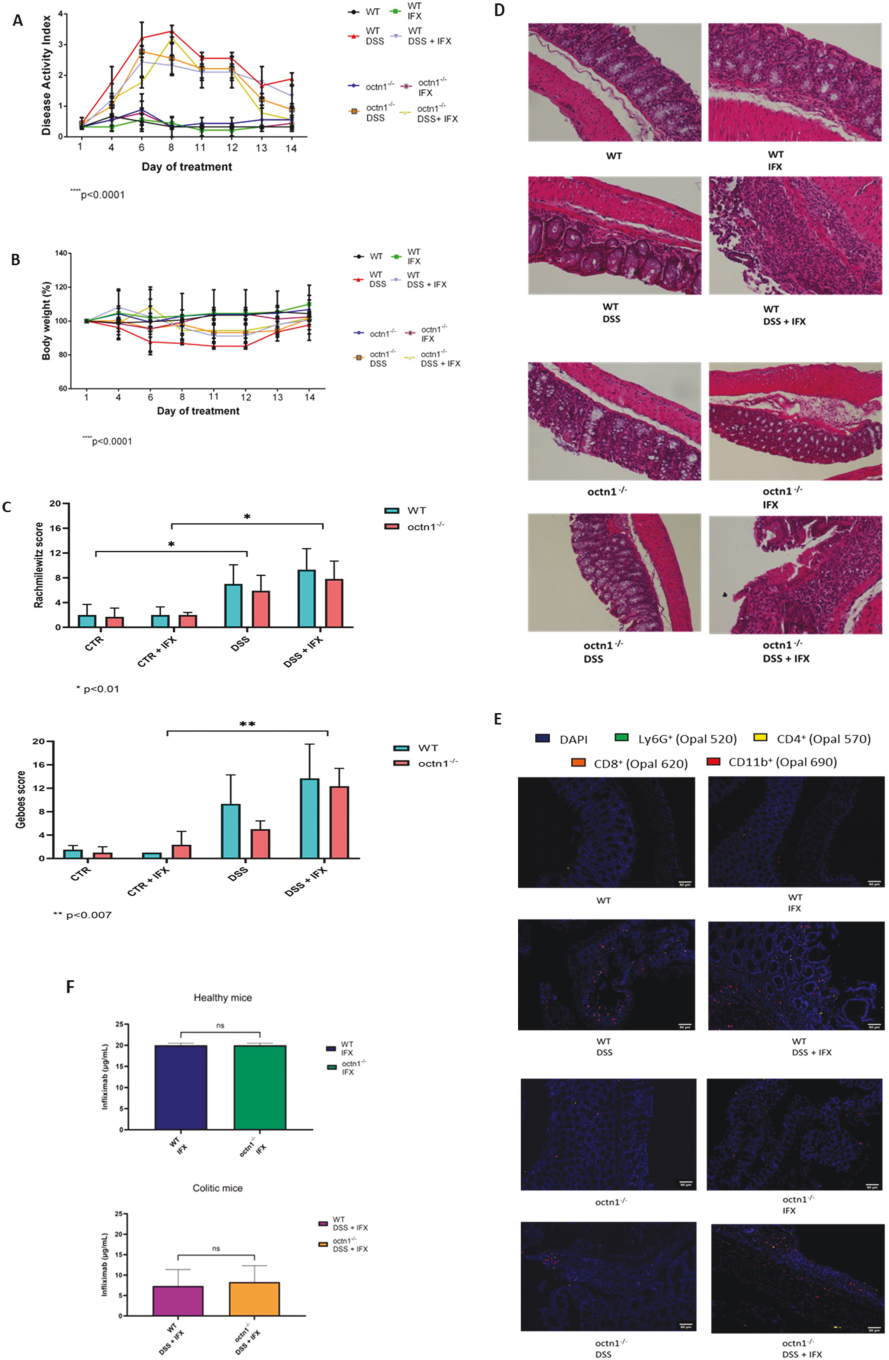
Differential response to DSS colitis and IFX in WT and *octn1*^-/-^ mice. Panels A and B: Four-point disease activity index (DAI) and body weight loss represented separately as percentage of day 1, over the entire treatment period. Traces and symbols corresponding to the eight treatment groups are indicated. Values are mean ± SD of 4 mice (per group). Panel C: Rachmilewitz and Geboes score (see Methods section) in WT and *octn1*^-/-^ treated mice. Data are represented of at least 3 independent experiments. Panel D: 20X Representative figures of colon (hematoxylin and eosin) in WT mice (upper panels) and in *octn1*^*-/-*^ mice (lower panels) with no treatment, DSS or treated by IFX. Panel E: Representative images of multiplex immunofluorescence staining. The panel displays multiplex IHC 8 images after spectral unmixing, of LY6G + neutrophils (green), CD8 + cytotoxic T cells (orange), CD4 + T helper cells (yellow), CD11b + granulocytes (red), and a nuclear marker (DAPI, blue). Scale bars, 50 μm. Panel F: Serum concentration of IFX in healthy and DSS-treated WT and *octn1*^*-/-*^ mice. Blood samples were collected at sacrifice. Infliximab was quantified by ELISA (Sanquin, the Netherlands) according to the manufacturer’s recommendations. Values are mean ± SD of 4 mice (per group).

*Octn1* ^*-/-*^ mice displayed a milder course of colitis compared with wild type controls, as shown by a lower DAI and a less severe body weight loss; however, both DSS-treated strains displayed colon reddening and shortening at sacrifice (Figure 1 panel A; Supplementary Figure 2). The response to IFX also differed between the 2 murine strains, being the drug more effective in wild type than in octn1^-/-^ mice in attenuating body weight loss and colitis activity (Figure 1 panel B). Histological scoring of hematoxilyin-eosin stained intestinal sections, performed according to Rachmilewitz and Geboes, confirmed milder lesions in *octn1*^*-/-*^ animals. Unexpectedly, the same analysis revealed a clear trend towards worsening inflammation in the IFX-treated WT groups and, again, no or limited response to the drug in the transporter-deficient strain (Figure 1, panels C and D). These differences were also mirrored by multiplex IHC staining of colonic mucosa for different inflammatory cell populations (Figure 1, panel E). Importantly, the serum concentration of IFX did not differ between the 2 strains (Figure 1, panel F).

### *Octn1* ^-/-^ Influences Microbiota Profile

**Figure 2.**
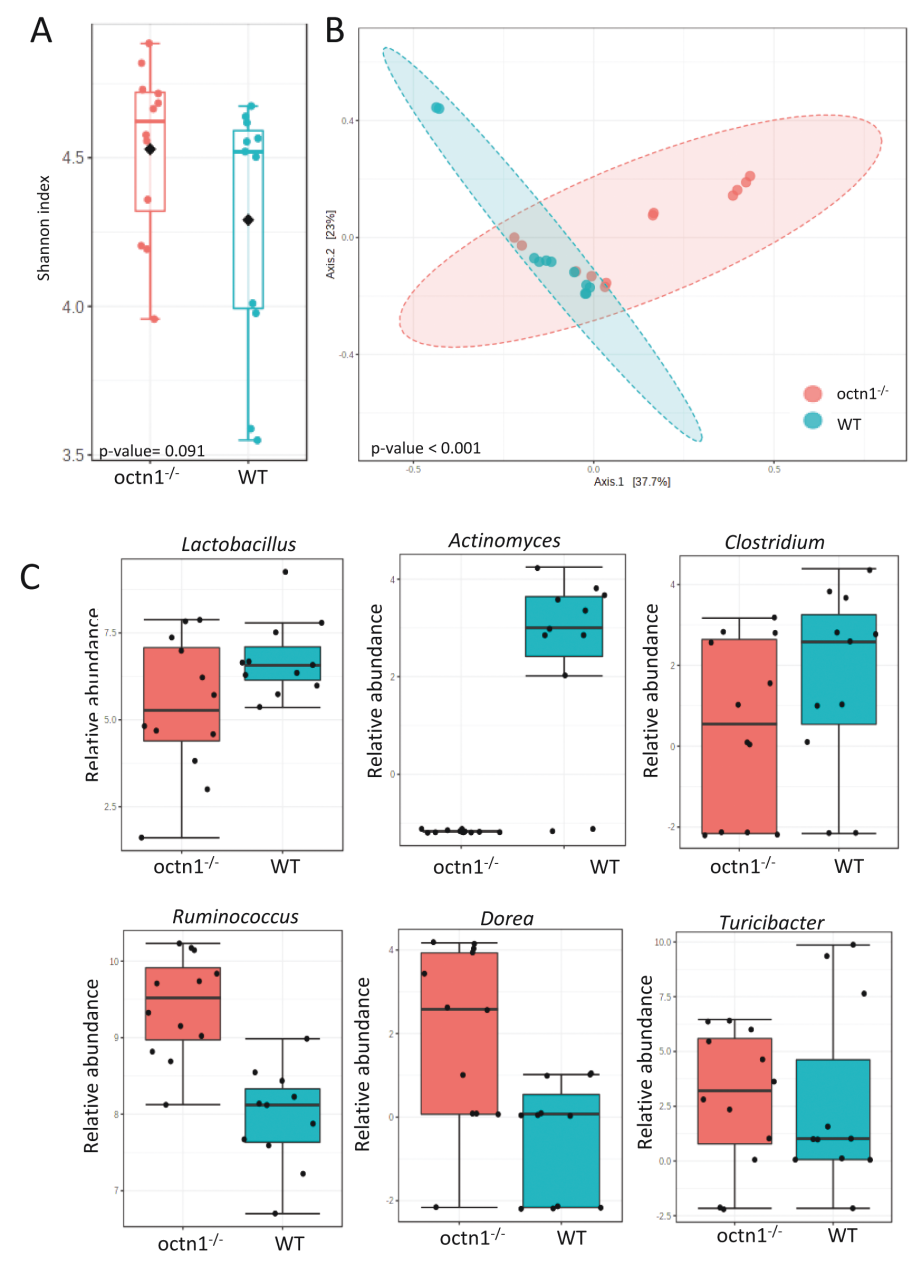
*octn1* ^-/-^ influenced microbiota profile. Panel A: Shannon index box plots calculated on gut microbiota of *octn1*^-/-^ and WT mice. *P* value, corrected for FDR, was determined by Mann-Whitney test. Panel B: Principal Coordinates Analysis (PCoA) plot of Bray Curtis dissimilarity matrix of *octn1*^-/-^ and WT animals. *P* value is by PERMANOVA test. Panel C: Relative abundance of different bacterial phyla in *octn1*^-/-^ and WT; comparisons were by DESeq2 statistics (*P* value FDR < .05).

Ecological analyses of fecal samples showed richness and evenness of gut microbiota in *octn1*^*-/-*^ mice strain in respect with WT (Figure 2, panel A).

Likewise, to detect specific changes in the gut microbial ecosystem in the context of *octn1* deletion, we calculated β-diversity by Bray-Curtis metrics. This analysis highlighted a significant intragroup clusterization for both *octn1*^-/-^ and WT samples (PERMANOVA *P* < .001; Figure 2, panel B). Moreover, we observed a statistical increase of *Lactobacillus*, *Actinomyces* and *Clostridium* in the WT group, while *Ruminococcus*, *Dorea*, and *Turicibacter* were increased in *octn1*^-/-^ group (Figure 2, panel C).

### Microbial Signatures of Colitis-induced Dysbiosis in WT and *octn1*^-/-^ Mice

**Figure 3.**
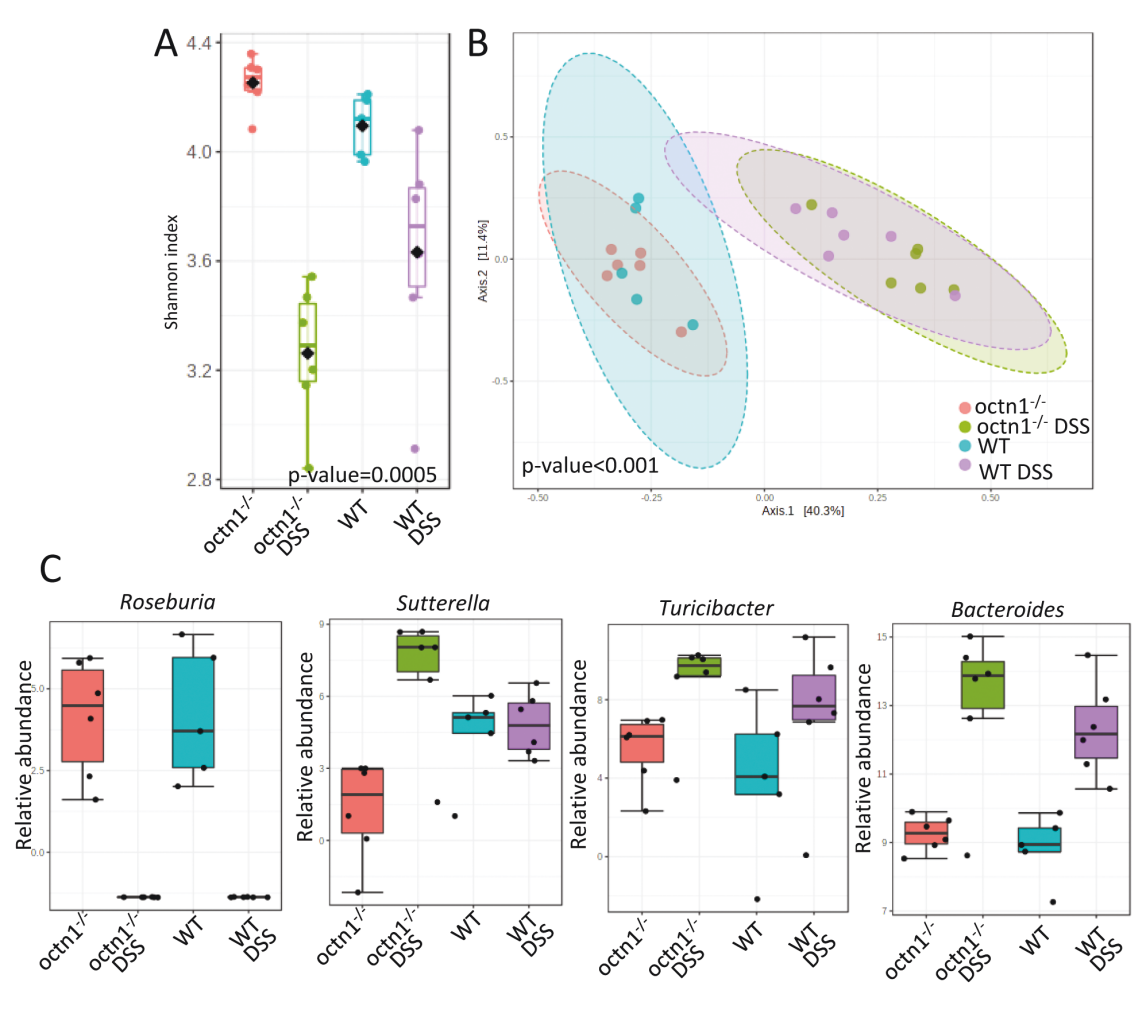
Colitis-induced gut microbiota dysbiosis in WT and *octn1*^-/-^ mice. Panel A: Shannon index box plots calculated on gut microbiota of *octn1*^-/-^ and WT animals in presence or not of DSS induced colitis. *P* value, corrected for FDR, has been obtained by Kruskal-Wallis test. Panel B: PCoA plot of Bray Curtis dissimilarity matrix of *octn1*^-/-^ and WT genotypes in presence or not of DSS induced colitis. *P* value has been computed by PERMANOVA test. Panel C: Relative abundance of the indicated phyla by mouse strain and experimental treatment. Comparisons among the 4 groups (*octn1*^-/-^ and WT with or without DSS) were by DESeq2 statistics.

The DSS-induced colitis produced a profound dysbiosis, marked by decreased gut microbiota richness in both treated strains compared with nontreated animals (Figure 3, panel A).

Beta diversity showed that samples from DSS-treated WT and octn1^-/-^ mice clustered together with respect to untreated mice (Figure 3, panel B). *Roseburia* was completely reduced in the presence of colitis in both strains, while *Sutterella, Turicibacter*, and *Bacteroides* were higher in the *octn1*^-/-^ DSS treated group compared with others (Figure 3, panel C). The heatmap, based on relative abundances of bacteria, revealed the increase of taxa clustered in cluster 1 (Ruminococcaceae, *Oscillospira*, *Dehalobacterium*, *Bilophila*, *Odoribacter*, *Ruminococcus*, Erysipelotrichaceae, *Anaeroplasma*, Clostridiaceae, *Coprococcus*, Lachnospiraceae and *Prevotella*) in WT mice treated by DSS. The cluster 2 was composed by *Sutterella*, *Parabcteroides*, S24_7, Lachnospiraceae Ruminococcus, *Bacteroides*, *Turicibacter*, which were increased in *octn1*^-/-^ mice treated with DSS. Finally, the cluster 3 included Enterobacteriaceae, F16, *Lactobacillus*, Prevotellaceae, *Prevotella*, *Roseburia*, *Mucispirillum*, Rikenellaceae, high in WT and *octn1*^-/-^ mice without colitis (Supplementary Figure 3, panel A).

### IFX Treatment Attenuates Colitis-associated Dysbiosis

**Figure 4.**
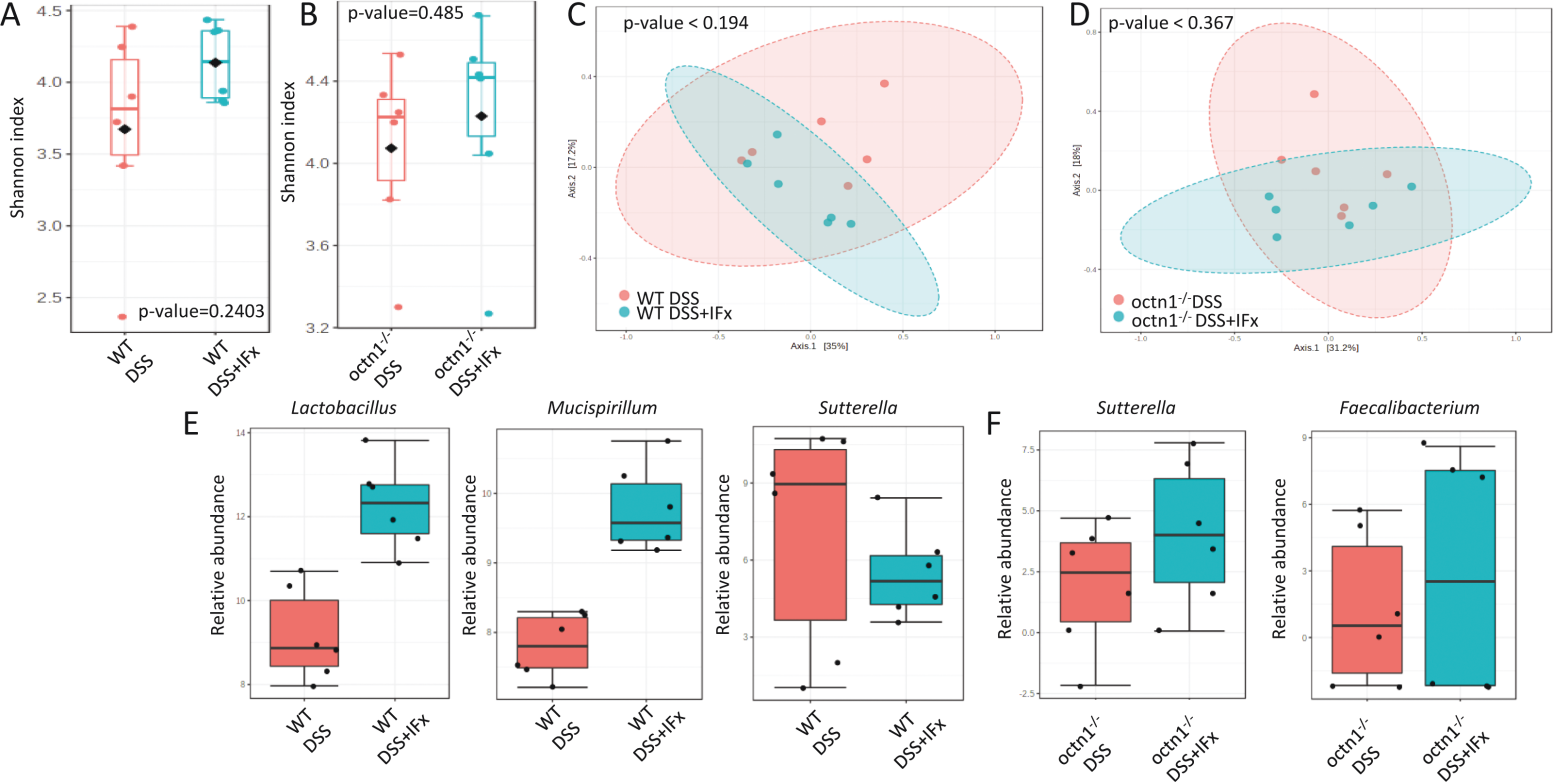
Infliximab improves dysbiosis in the presence of colitis. Panels A and B: Shannon index box plots illustrating preserved microbial alpha diversity in colitis-bearing WT and *octn1*^-/-^ mice treated with IFX. *P* values, corrected for FDR, have been obtained by Mann-Whitney test. Panels C and D: PCoA plots of Bray Curtis dissimilarity matrix showing lack of sample clustering by IFX treatment. *P* values have been computed by PERMANOVA test. Panels E and F: Relative abundance comparisons between DSS-treated mice for both strains with or without IFX. Statistics by the DESeq2 method.

Having observed differences in gut microbiota response to colitis between WT and *octn1*^-/-^ mice, we focused on the effect of IFX in the 2 strains. Infliximab treatment produced the expected increase of bacterial richness and evenness (*ie*, Shannon index) in WT and *octn1*^-/-^ mice (Figure 4, panels A and B). However, β-diversity analysis did not reveal an apparent clustering of samples due to IFX treatment (PERMANOVA *P* > .05; Figure 4, panels C and D).

In the WT group, the administration of IFX was associated with increased representation of *Lactobacillus* and *Mucispirillum*, and with lower relative abundance of *Sutterella* (Figure 4, panel E). Conversely, *Sutterella,* together with *Faecalibacterium,* was increased by IFX in *octn1*^-/-^ animals (Figure 4, panel F).

When the 2 DSS + IFX groups were directly compared with each other, a slightly higher value of Shannon index was noted in *octn1*^-/-^ mice (Supplementary Figure 4, panel A). Moreover, beta diversity analysis showed 2 distinct profiles associated with genotypes in response to IFX (Supplementary Figure 4, panel B). In fact, under IFX treatment, *octn1*^-/-^ mice showed a higher abundance of *Prevotella* and *Faecalibacterium,* and a reduction of *Bacteroides* compared with WT (Supplementary Figure 4, panel C).

Moreover, heatmap analysis revealed that taxa included in cluster 1 (*Sutterella*, Lachnospiraceae_*Rumnococcus*, Clostridiaceae, SMB53, Lachnospiraceae, *Anaerotruncus*, *Parabacteroides*, Peptococcaceae, *Dehalobacterium*, *Bilophila*, Ruminococcaceae, and *Oscillospira*) and 2 (*Prevotella*, *Lactococcus*, *Mucispirillum*, *Anaeroplasma*, *Coprococcus*, *Ruminococcus*, *Lactobacillus*, Mogibacteriaceae, *Odoribacter*, *Turicicbacter*, *Prevotella*, Christensellaceae, and Erysipelotrycaceae) were overall more represented in WT than in *octn1*^-/-^ mice under anti-TNFα therapy (Supplementary Figure 3, panel B).

### Microbial Functional Profiling

To infer functional modifications of gut microbiota associated with *octn1* status and IFX treatment, we generated KEGG pathway predictions by PICRUSt (Supplementary Figure 5). In the absence of colitis, the *octn1*^-/-^ microbiome was enriched in KEGG pathways for cellular processes, as bacterial flagellar assembly, chemotaxis, and sporulation and for glycerolipid metabolism. In the presence of colitis, *octn1*^-/-^ microbiome displayed a higher representation of metabolic pathways (degradation of glycan, glycosaminoglycan, and 1,1,1-Trichloro-2,2-bis [4-chlorophenyl] ethane [DDT]; metabolism of galactose, nitrogen, sphingolipid, cyano-amino acid, and pyruvate; biosynthesis of lipopolysaccharide) and of inorganic ion transport, metabolism, and restriction enzymes. In healthy WT animals, hyper-represented microbial pathways included bacterial motility proteins and RIG-I-like receptor signaling, while in presence of colitis only amino sugar and nucleotide sugar metabolism were enriched (Supplementary Figure 5, panel A).

When DSS + IFX (ie, colitis + infliximab) groups of different strains were compared, 14 metabolic pathways were enriched in *octn1*^*-/-*^ mice, including amino acid, carbohydrate, thiamine, sphingolipid, cyano-amino acid metabolism, glycan and phenylpropanoid biosynthesis, and 2 of photosynthesis. Secretion system and butanoate metabolism were instead over-represented in the WT strain (Supplementary Figure 5, panel B).

### *Octn1* ^-/-^ Mice Are Characterized by Differential Expression on FOXP3^+^CD25^+^CD4^+^Cells

Given the well-established role of Tregs in regulating inflammation, we investigated this specific T lymphocyte subset in WT and *octn1*^-/-^ mice across the different experimental conditions.

**Figure 5.**
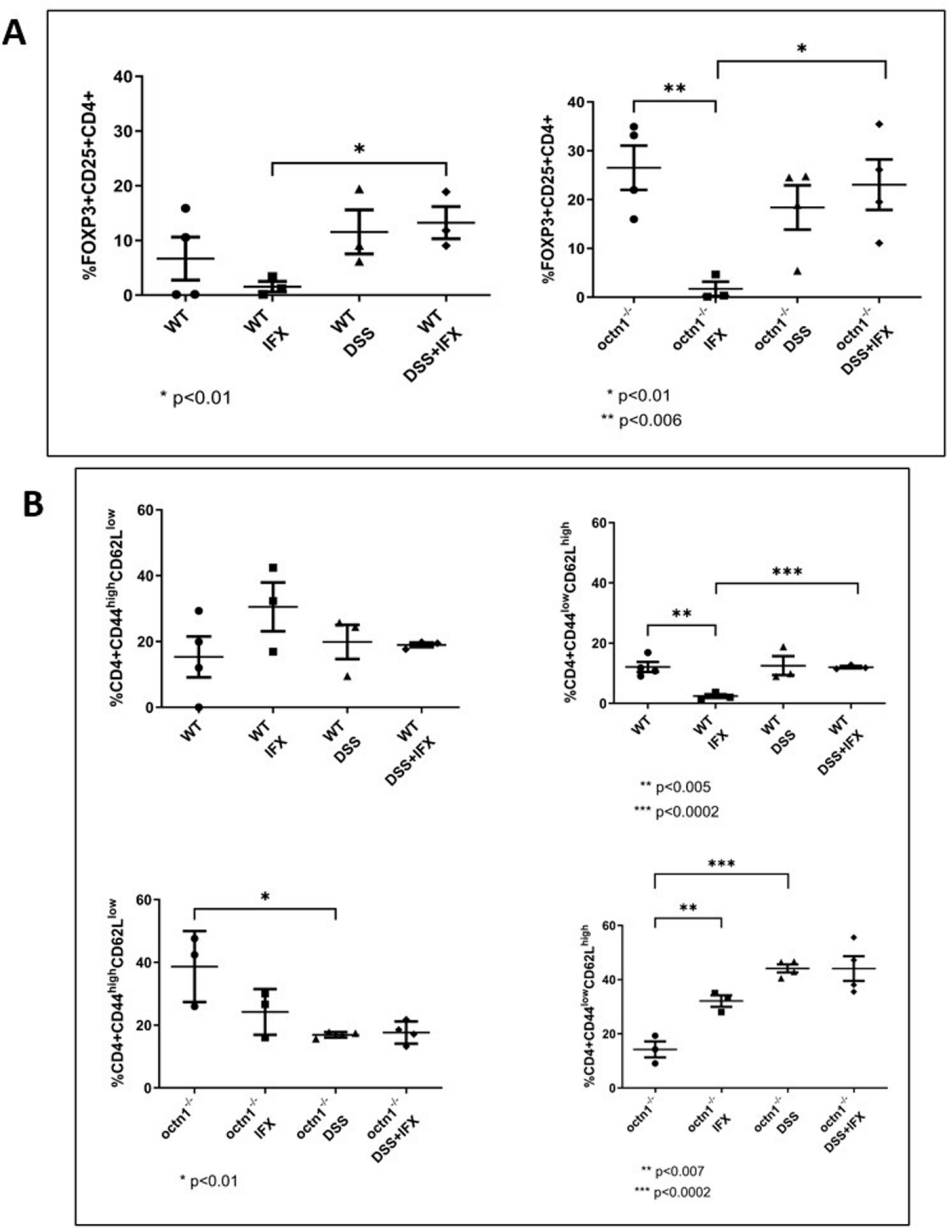
T-Reg, memory and naïve T cells populations count differences in *WT and octn1*^*-/-*^*mice*. Panel A: Dot plots representing the Treg populations (FOXP3+, CD25 + and CD4+) in the different treatment groups: WT/octn1^-/-^, WT IFX/octn1^-/-^ IFX, WT DSS/octn1^-/-^ DSS, WT DSS + IFX/octn1^-/-^ DSS + IFX. The graph on the left refers to the wildtype (WT) group and the graph on the right to the knockout group (*octn1*^-/-^). Data presented as mean ± SD of 4 mice (per group). Pictures in Wisker plot are representative of at least 3 independent experiments. Panel B: Dot plots show the distribution of memory (CD4 + CD44^high^CD62L^low^) and naïve (CD4 + CD44^low^CD62L^high^) lymphocytes in the different treatment groups: WT/octn1^-/-^, WT IFX/octn1^-/-^ IFX, WT DSS/octn1^-/-^ DSS, WT DSS + IFX/octn1^-/-^ DSS + IFX. Upper panels: WT; lower panels: *octn1*^**-/-**^. Data displayed as mean ± SD of 4 mice (per group). Pictures in Wisker plot are representative of at least 3 independent experiments.

As displayed in Figure 5 panel A, the Treg (*FOXP3*^*+*^*CD25*^*+*^*CD4*^*+*^) population was markedly expanded at baseline in *octn1*-deficient animals compared with WT controls. This difference was less pronounced in the treated groups, with IFX drastically reducing Tregs in both strains, and DSS or DSS + IFX increasing their number over the baseline in WT while lowering their percentage in octn1^-/-^.

These cells were reduced in the group treated with *octn1*^-/-^ DSS relative to the corresponding control, while being increased in the WT DSS-treated group.

We also analyzed the Treg populations not expressing CD25 and expressing interleukin-17 (FOXP3+, CD4+, CD25- and IL17+) and the Treg expressing interleukin-17 (FOXP3+, CD4+, CD25 + and IL17+). The results showed no changes in relation to the strain (Supplementary Figure 6).

### The *octn1*^-/-^ Genotype Affects Enteric Memory and Naïve T Lymphocytes

To further characterize the immunological phenotype across the strain and experimental treatments, we quantified by FACS memory (CD4 + CD44^high^CD62L^low^) and naïve (CD4 + CD44^low^CD62L^high^) T lymphocytes from mesenteric lymph nodes. At baseline, *octn1*^-/-^ mice displayed a higher percentage of memory lymphocytes compared with wild type, and IFX nearly abolished this difference. Remarkably, in *octn1* deficient mice, memory and naïve T cells displayed an opposite response to colitis induction, with the former population decreasing and the latter increasing in the DSS and DSS + IFX groups (Figure 5, panel B—lower panels). Conversely, in the WT mice group, the percentage of naïve and memory lymphocytes remained relatively unaffected by experimental colitis; (Figure 5, panel B—upper panels). Infliximab treatment after DSS had no major effect in either mouse strain. Thus, collectively, these aforementioned changes highlight a marked difference in enteric immune cell phenotype and immunological response to colitis in animals lacking the OCTN1 transporter.

### *Octn1* ^-/-^ Genotype Is Associated With Increased T-Helper Type 17 (Th17) Counts at Baseline

To gain more information on how *octn1* could affect T helper function, we evaluated the percentage of Th1, Th2 and Th17 cells in mesenteric lymph nodes from *octn1*^-/-^ and WT animals.

**Figure 6.**
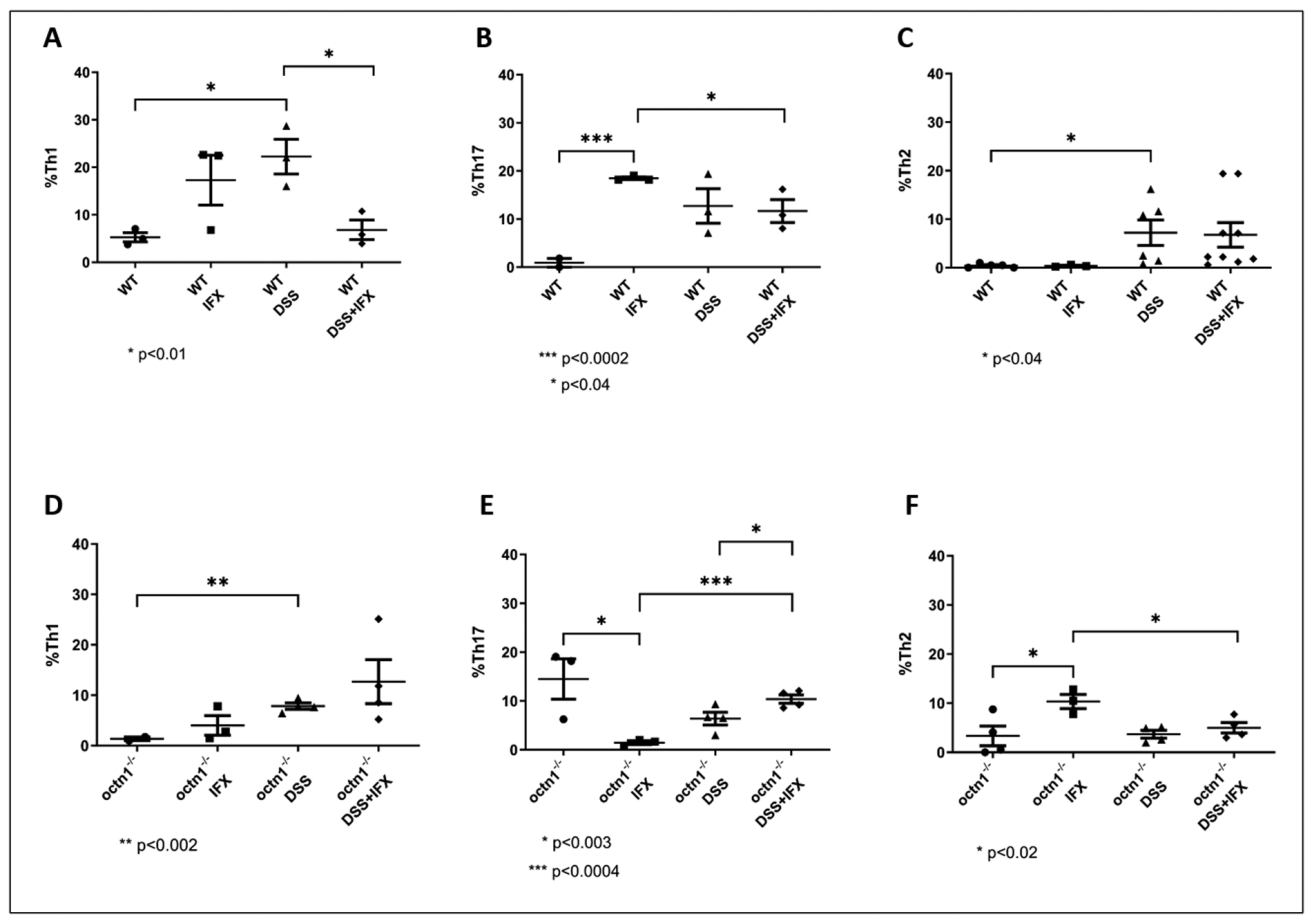
T-helper populations in *WT* and *octn1*^*-/-*^ mice. Dot plots show the distribution of T-helper type 1 (Th1), T-helper type 17 (Th17) and T-helper type 2 (Th2) lymphocytes in the different treatment groups: WT/octn1^-/-^, WT IFX/octn1^-/-^ IFX, WT DSS/octn1^-/-^ DSS, WT DSS + IFX/octn1^-/-^ DSS + IFX. Upper panels: WT; lower panels: *octn1*^**-/-**^. Data are displayed as mean ± SD of 4 mice (per group). Pictures in Wisker plot are representative of at least 3 independent experiments.

In the WT group, the levels of these 3 T-helper subtypes increased with induction of colitis compared with healthy untreated controls (Figure 6, upper panels). Infliximab failed to lower Th17 and Th2 levels after colitis (Figure 6, panels B and C), but it did decrease Th1 lymphocyte numbers, returning them to near baseline levels (Figure 6, panel A).

Conversely, in *octn1*^-/-^ mice, we noted an increase in Th17 at baseline, which was significantly reduced by DSS-induced colitis (Figure 6, panel E). The Th1 and Th2 were instead increased in colitis-bearing mice, although to a lesser extent compared with WT (Figure 6, panels D and F).

Infliximab treatment after DSS administration did not have major effects on T-helper lymphocytes, except for Th1 (increased in this strain while decreased in WT; Figure 6, lower panels). Notably, IFX alone induced Th17 in WT, while drastically reducing the same population in *octn1*^-/-^ mice. Although mechanistic explanations for these changes remain elusive, this observation confirms the profound alteration of the enteric immune phenotype associated with the loss of OCTN1 function.

## Discussion

In this study, we evaluated the effect of the transporter OCTN1 on gut microbiota and mucosal T cell populations in experimental colitis and response to IFX treatment.

To evaluate the impact of OCTN1 on intestinal microbiota and its colitis-associated modifications, we took advantage of a KO mouse strain originally generated by Kato et al.^9^*Octn1*^*-/-*^ mice displayed a milder disease compared with WT after induction of colitis with DSS, with a lower DAI score for the entire duration of the treatment and less severe mucosal damage and inflammatory infiltration at sacrifice. Interestingly, clinical response to IFX was also attenuated in KO mice compared with their controls, involving OCTN1 in the mechanism of action of the drug. Of note, IFX paradoxically exacerbated mucosal inflammation in WT and, although to a lesser extent, in transporter-deficient animals. While this unexpected finding is in line with a similar observation in DSS-treated TNF-α^-/-^ compared with TNF- α^+/+^ mice,^30^ it should also be noted that in our model, mice were killed not at the peak of clinical disease but after a 1-week recovery, when histological evidence of increased leukocyte infiltration may signal a better and faster tissue repair. Additionally, systemic anti-inflammatory effects of TNF blockade by IFX may contribute to the observed misalignment between clinical and histological responses to the drug.

In our experimental settings, the induction of colitis impacted intestinal dysbiosis more than *octn1* mutation. In fact in both strains, *Roseburia* was markedly reduced, and *Turicibacter* and *Bacteroides* were increased. Reduced abundance of *Roseburia* has already been reported in the inflamed gut of UC patients,^31^ while the increase of *Turicibacter* and *Bacteroides* was correlated with the induction of gut inflammation in animals treated by DSS.^32^

However in WT mice, the administration of IFX produced a positive beneficial effect marked by the increase of the probiotic *Lactobacillus* and a decreased representation of the inflamogenic *Sutterella;* in *octn1*^-/-^, instead, the effect of IFX was arguable, with a concomitant increase of the beneficial *Faecalibacterium* and the harmful *Sutterella*. The role of *Sutterella* in gut inflammation has been reported in UC patients, in which a decrease of this microorganism was correlated with a corticosteroid-free remission of the disease.^33^ However, when we compared the response to IFX treatment between *octn1*^-/-^ and WT mice during colitis, we observed an increase of bacterial richness and of *Prevotella* and *Faecalibacterium* in *octn1*^-/-^. Again, we highlighted a nonlinear effect of IFX in *octn1*^-/-^. In fact in mice, previous studies have correlated *Prevotella* with high susceptibility to chemically induced colitis^34^ and to mucosal inflammation and intestinal dysbiosis.^35^ On the other hand, the increase of *Faecalibacterium* has been detected in the intestinal microbiota of CD patient responders to anti-TNFα.^36,37^

In *octn1*^*-/-*^ in colitis condition, the KEGG pathways’ predictions revealed a significant number of inflammatory pathways involved in molecule degradation, inflammatory processes, and lipopolysaccharide biosynthesis. Instead, IFX administration increased the pathways of amino acid metabolism, which are the precursors for the bacterial synthesis of the anti-inflammatory short chain fatty acids (SCFAs).

In the gut, the microbiota and immune cells constantly interact and profoundly influence each other to maintain intestinal homeostasis.^38^ We initially focused on Tregs, as per their role in maintaining immune homeostasis and establishing tolerance to nonpathogenic foreign antigens, including those found in commensal bacteria and food.^39^ The *octn1*^-/-^ mice had a higher baseline percentage of Treg than the WT, while in the treated groups the percentage of Treg was comparable in the 2 strains. This finding, together with information from microbiota analysis (high α-diversity but also more pathogenic species represented) and metabolic pathway prediction (linked to bacterial virulence factors’ expression as flagellar assembly, chemotaxis and sporulation) suggests *octn1*^*-/-*^ mice experienced in basal conditions, an exacerbated immune challenge, and possibly the development of immune exhaustion or an active suppression mechanism of the immune response via Tregs. Such chronic overstimulation of adaptive immunity may also reflect a primitive defect in bacterial sensing and inflammatory responses of innate immune and antigen-presenting cells, or a leaky epithelial barrier. Importantly, this view is perfectly consistent with the lower DAI score in the *octn1*^-/-^ compared with the WT under colitis induction and the somewhat blunted response to anti-TNFα. Moreover, this interpretation is further supported by our findings of constitutively elevated numbers of memory (CD4 + CD44^high^CD62L^low^) T lymphocytes in mesenteric lymph nodes of *octn1*-deficient mice, suggestive of an ongoing intestinal immune response even before chemical challenge. Also of note, Th2 and Th17 lymphocytes, most directly linked to IBD pathogenesis,^40^ were higher at baseline and reduced after DSS in *octn1*^-/-^ mice compared with WT, again consistent with a preexisting expansion but reduced responsiveness of these T helper subpopulations in the context of OCTN1 deficiency.

We are aware of a few limitations of the study. One first limit lies in the choice of *octn1*^**-/-**^ model; since *octn1* is ubiquitously deleted in these mice, the cell compartment from which the transporter most critically influences gut inflammation (ie, epithelial cells, inflammatory cells or both) remains to be clarified. Colocalization experiments showed a marked overlap between OCTN1 and CD68, a macrophage marker, in the mucosal interstitium of DSS-treated WT animals (Supplementary Figure 7), with minimal positivity of epithelial cells for the transporter. However, while a predominant role of the carrier in inflammatory cells is conceivable, this aspect deserves further investigation. Similarly, we cannot exclude extraintestinal influences on the severity of the gut inflammatory phenotype. Additionally, by being *octn1* completely absent in these mice, the model is only partially representative of the increased risk of IBD associated with the L503F variant in humans. On the other hand, our observation of attenuated intestinal inflammation in the absence of *octn1* may suggest a gain-of-function role for this risk allele in human disease. Additional limitations related to microbiota studies are the low number of fecal samples for each studied condition and the resort to metabolic pathways prediction as a surrogate for the direct measurement of metabolic stool content.

Notwithstanding these shortcomings, our data strongly argue in favor of an essential causative role of OCTN1 in the complex and multifactorial pathogenesis of IBD. More specifically, the findings presented here place OCTN1 in the center of an intricate and still incompletely elucidated pathogenic network in which microbe-host interactions, dysbiosis, and deregulated adaptive mucosal immunity reciprocally influence each other. While the mechanistic underpinnings of this circuitry await further investigation, our work entails important translational implications: on the one hand, we suggest that *octn* genotyping in IBDs patients combined with microbiota profiling could help monitor the progression of the disease and predict the individual response to therapy; moreover, by reinforcing the notion that OCTN1 participates as a causative or predisposing factor to IBD, our work lays the groundwork for novel targeted therapies aimed against this carrier and/or its transported substrates.

## Supplementary Data

Supplementary data is available at *Inflammatory Bowel Diseases* online.

izae135_suppl_Supplementary_Figures_1-7

## Data Availability

Datasets will be shared upon request.
