# Supplementary material for: Solute Transporter OCTN1/Slc22a4 Affects Disease Severity and Response to Infliximab in Experimental Colitis: Role of Gut Microbiota and Immune Modulation
Source: Inflamm Bowel Dis. 2024 Jun 29;30(12):2259–70. doi: 10.1093/ibd/izae135 (PMC11630256; doi:10.1093/ibd/izae135)
Supplement: izae135_suppl_Supplementary_Figures_1-7 [file izae135_suppl_supplementary_figures_1-7.docx]

**Supplementary Materials**


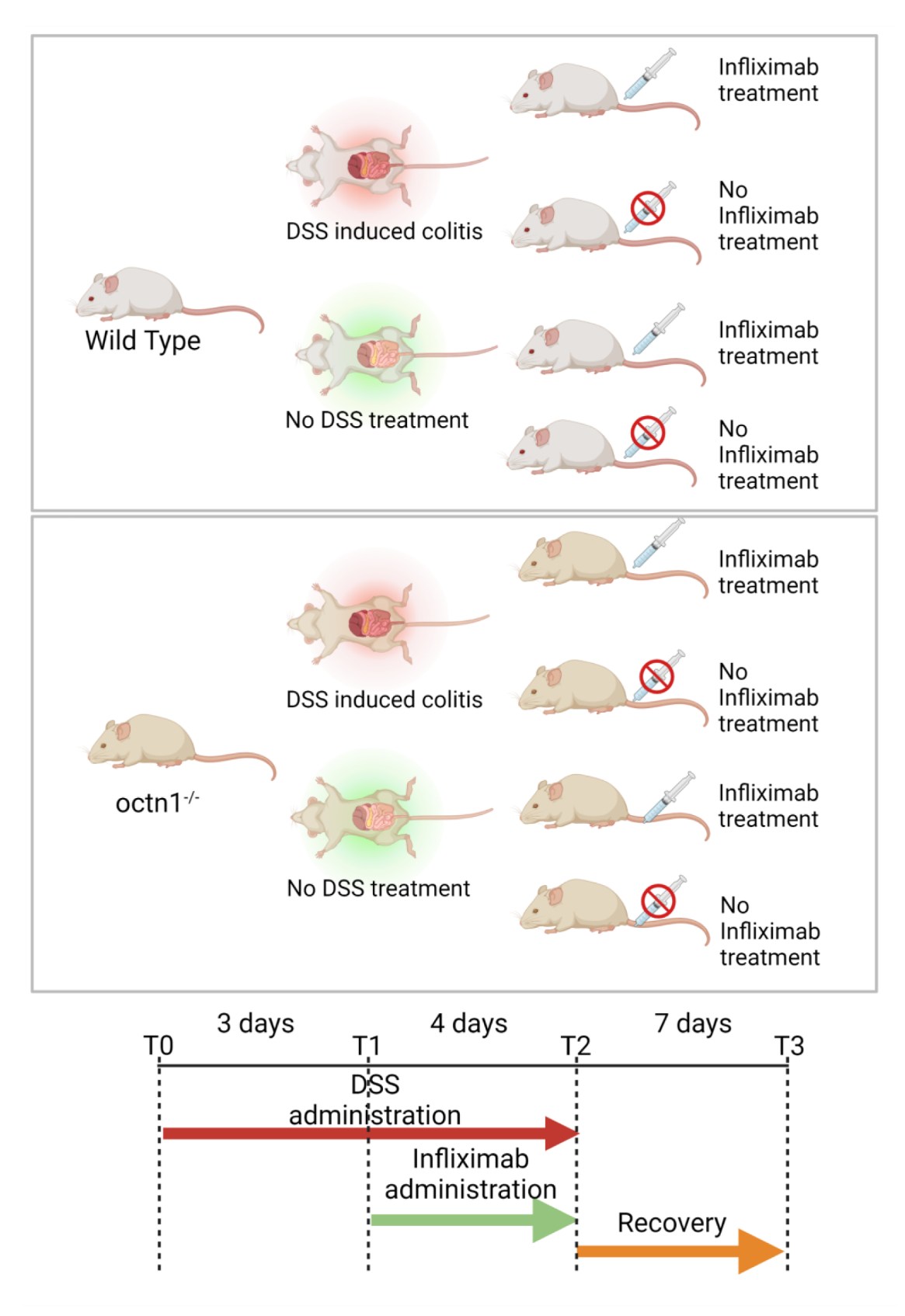


**Supplementary Figure 1.** Schematic representation of animal experimental workflow.


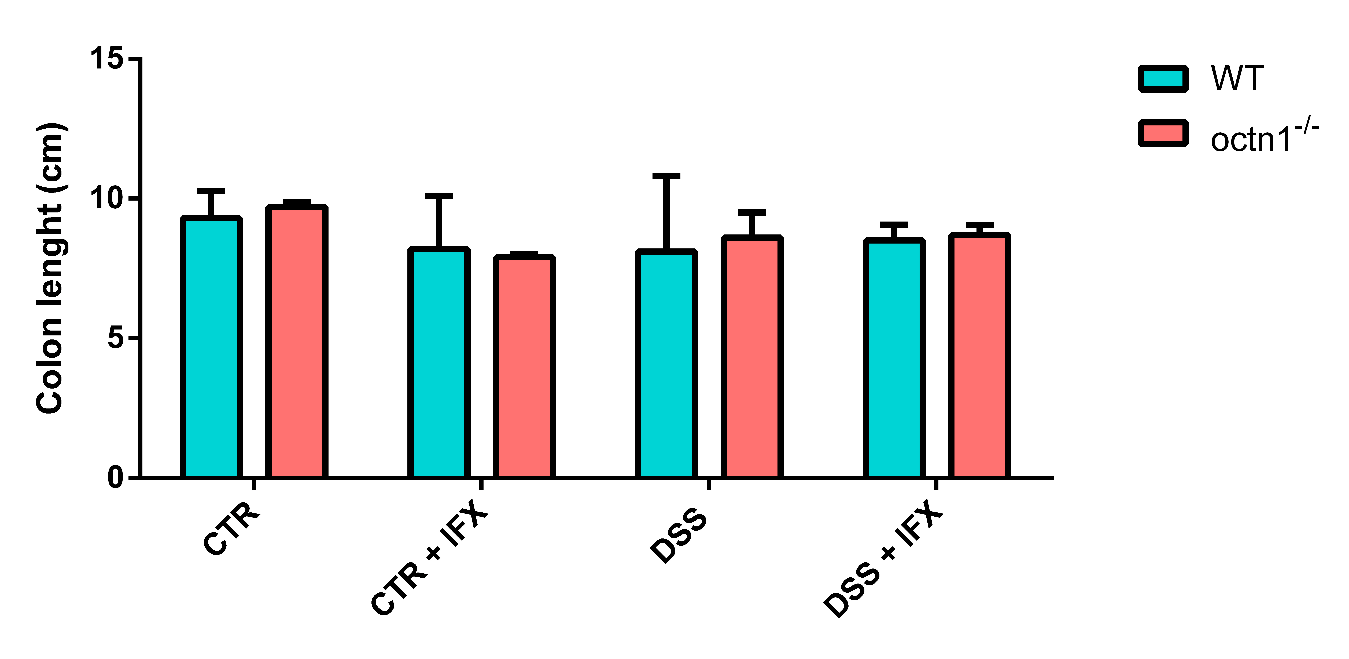


**Supplementary Figure 2.** Colons from DSS-treated mice displayed reddening and shortening, which are typical signs of acute intestinal inflammation. Values are mean±SD of 4 mice (per group). Data are displayed in bar plots and are representative of at least 3 independent experiments.


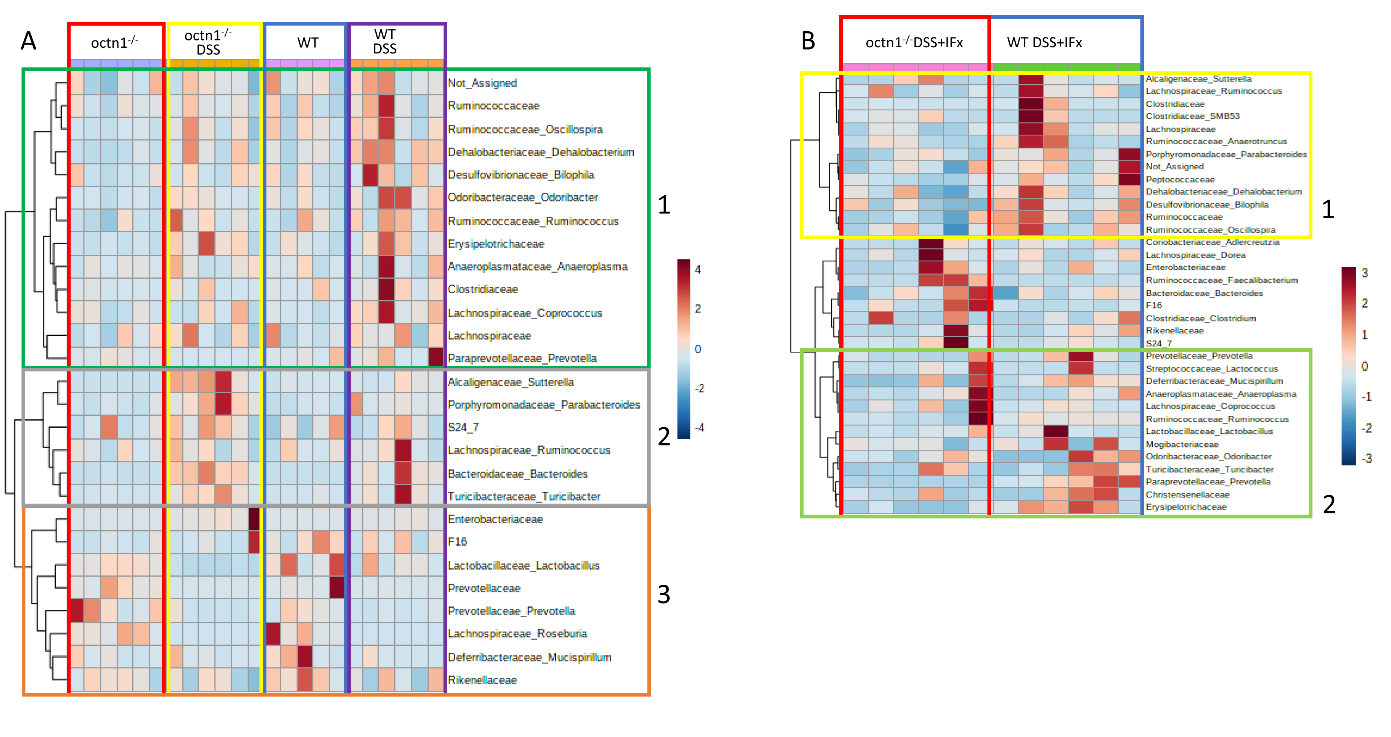


**Supplementary Figure 3.** Panel A: heatmap of gut microbiota profiles of *octn1^-/-^* and WT strains in presence or not of DSS induced colitis. Panel B: heatmap of gut microbiota profiles of WT and *octn1^-/-^* in presence of DSS-induced colitis and treated with IFX.


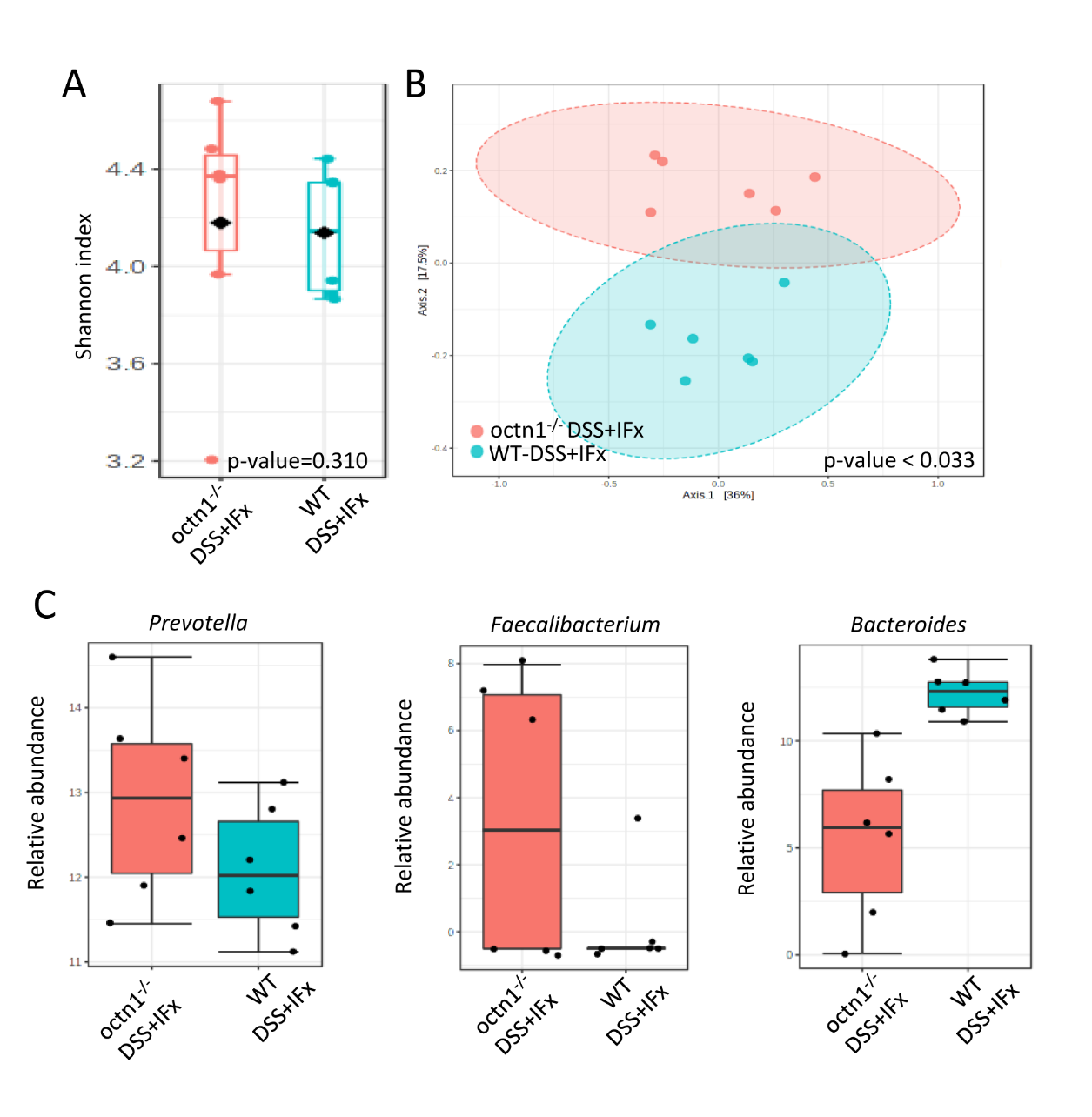


**Supplementary Figure 4.** OCTN1 genotype influences microbiota composition in response to IFX treatment. Panel A: Shannon index box plots calculated on the indicated experimental groups. P-values, corrected for FDR, have been obtained by Kruskal-Wallis test. Panel B: PCoA plot of Bray Curtis dissimilarity matrix of WT and *octn1*^-/-^ genotypes in presence of DSS induced colitis and IFX treatment. P-value has been computed by PERMANOVA test. Panel C: DESeq2 relative abundance comparison between WT and *octn1*^-/-^ mice treated with DSS+IFX Differentially abundant microbial genera are indicated.


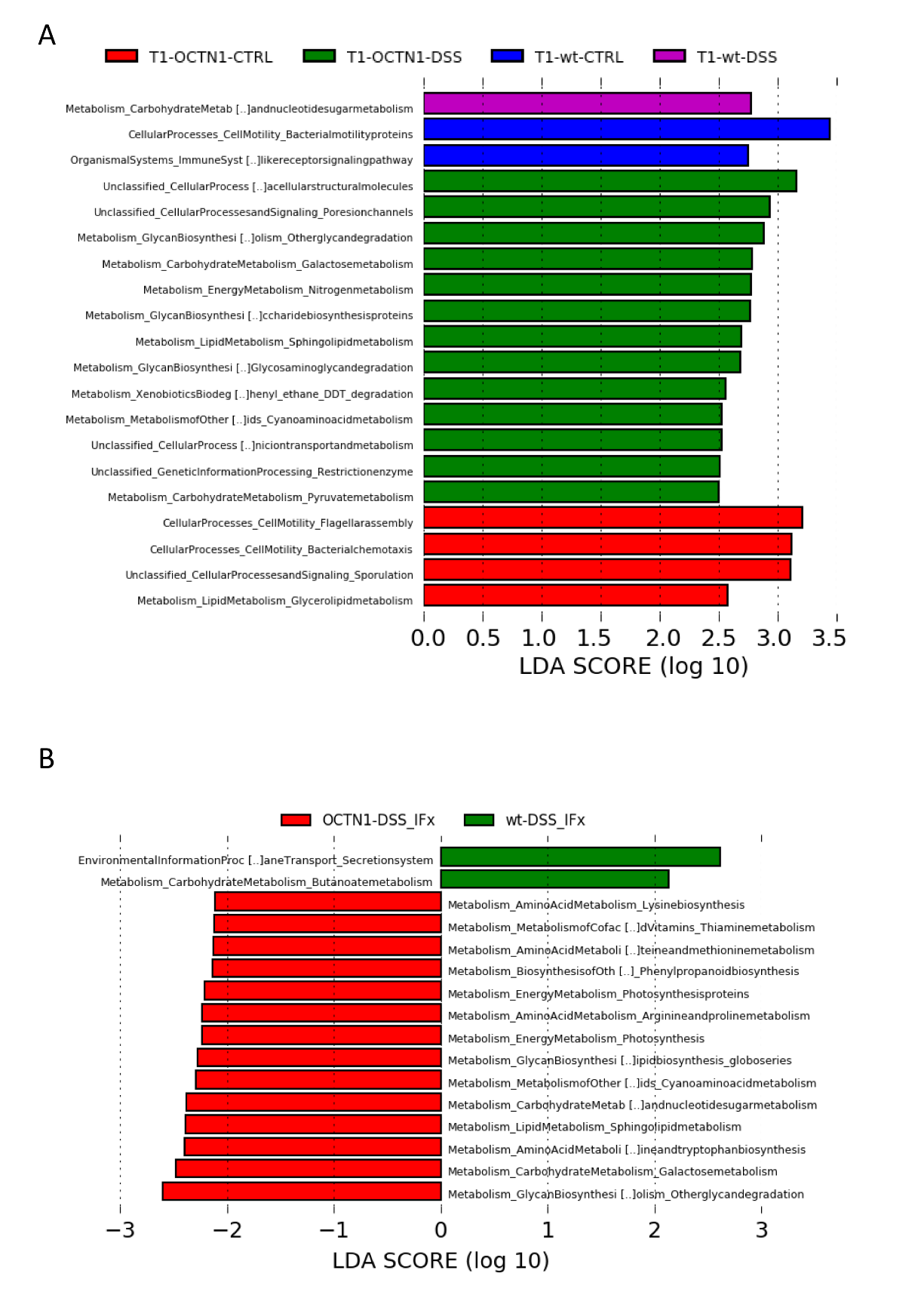


**Supplementary Figure 5.** Differentially abundant microbial pathways in Octn1 KO versus WT mice. PICRUSt-predicted KEGG pathways in *octn1*^-/-^ and in WT in presence or absence of colitis (A) or during IFX treatment (B). Linear discriminant analysis (LDA) effect size (LEfSe) was performed on the predicted KEGG pathways. Significance was set to ±2.0, and the log (10)-transformed score is shown to demonstrate effect size.





**Supplementary Figure 6.** Analysis of the CD25- (FOXP3+, CD4+, CD25- and IL17+) and CD25+ (FOXP3+, CD4+, CD25+ and IL17+) Treg populations.

Upper panels: WT; lower panels: *octn1***^-/-^**. Data presented as mean±SD of 4 mice (per group). Pictures in Wisker plot are representative of at least 3 independent experiments.


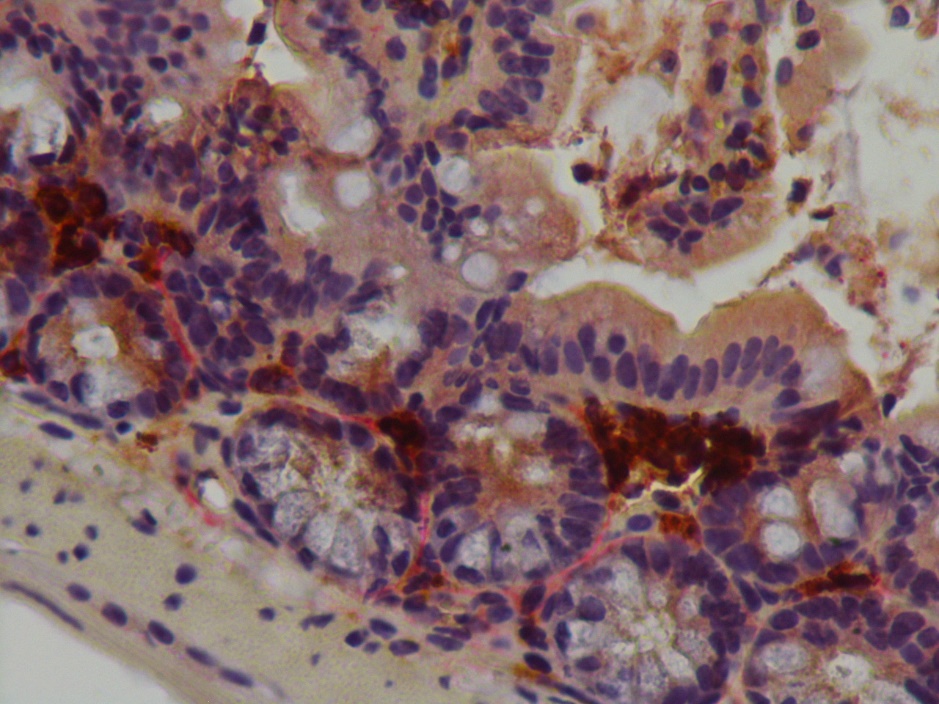


**Supplementary Figure 7.** Octn1 co-localizes with the macrophage marker CD68. 40X Representative immunohistochemistry of inflamed colonic mucosa double-stained for CD68-pgm1RTU(brown)/octn1(red), Immunostainer BOND MAX III (Leica).
